# The Scientific American

**Published:** 1881-12

**Authors:** 


*The Scientific American.*—It is almost unnecessary to do more than remind our readers of the commencement of a new volume of this widely known and useful publication, which finds a welcome in every professional and business man's office. A great amount of useful and highly interesting and practical matter is to be found in every weekly issue concerning Art, Science, Mechanics, Chemistry and Manufactures. The Publishers are Munn & Co., 37 Park Row, New York City, and the subscription is $3.20 per annum, postage prepaid. One copy six months $1.60.

The same publishers issue a supplement to the above, which is also a weekly, and a distinct paper, every number of which contains 16 octavo pages, uniform in size with the Scientific American. The subscription price is $5.00; or for the two publications seven dollars per annum.

